# Association between dietary intake and the expression of clock genes in adults: a brief report

**DOI:** 10.3389/fnut.2025.1663559

**Published:** 2025-10-08

**Authors:** Marlene Lages, Renata Barros, Marisa Ferreira-Marques, Joana Correia, Armando Caseiro, Maria P. Guarino, Sara Carmo-Silva

**Affiliations:** 1 ciTechCare – Center for Innovative Care and Health Technology, Polytechnic University of Leiria, Leiria, Portugal; 2 Faculty of Nutrition and Food Science, University of Porto, Porto, Portugal; 3 Laboratory for Integrative and Translational Research in Population Health (ITR), University of Porto, Porto, Portugal; 4 EPIUnit – Institute of Public Health, University of Porto, Porto, Portugal; 5 Coimbra Health School, Polytechnic University of Coimbra, Rua da Misericórdia, Lagar dos Cortiços, S. Martinho do Bispo, Coimbra, Portugal; 6 CNC-UC – Center for Neuroscience and Cell Biology, University of Coimbra, Coimbra, Portugal; 7 CIBB – Center for Innovation in Biomedicine and Biotechnology, University of Coimbra, Coimbra, Portugal; 8 Faculty of Pharmacy, University of Coimbra, Coimbra, Portugal; 9 H&TRC- Health and Technology Research Center, Coimbra Health School, Polytechnic University of Coimbra, Rua 5 de Outubro, Coimbra, Portugal; 10 University of Coimbra, CIPER, Coimbra, Portugal; 11 ESSLei, School of Health Sciences, Polytechnic University of Leiria, Leiria, Portugal

**Keywords:** chrononutrition, circadian rhythms, diet, obesity, clock genes

## Abstract

**Background:**

The circadian system regulates several physiological processes, including energy metabolism, through the expression of core clock genes. Animal studies suggest that dietary composition can influence circadian gene expression; however, evidence in humans remains limited.

**Objective:**

This study aimed to explore the association between dietary macronutrient intake and the expression of circadian clock genes in the whole blood of adults with distinct metabolic profiles.

**Methods:**

Nineteen adults (94.7% female; 43.4 ± 16.05 years) were categorized into healthy-weight and overweight/obesity groups based on BMI. Dietary intake was assessed using a 3-day food diary, and clock gene expression (*CLOCK*, *BMAL1*, *PER2*, *CRY*) was evaluated in whole blood samples collected between 08:00 a.m. and 04:00 p.m. Statistical analyses included group comparisons and correlation analyses between macronutrient intake and gene expression.

**Results:**

No statistically significant differences were observed in the clock gene expression between BMI groups, although group-level trends were noted, particularly in *PER2* and *CRY* expression. The overweight/obesity group exhibited a significantly higher percentage of energy intake from protein. Correlation analyses revealed several time-dependent associations between macronutrient intake and clock gene expression. Notably, in the healthy BMI group, *BMAL1* and *CRY* expression correlated with lipid and protein intake, while in the overweight/obesity group, *CLOCK* expression was strongly associated with both lipid and carbohydrate intake.

**Conclusion:**

These findings suggest that macronutrient composition may influence peripheral circadian gene expression in a time- and BMI-dependent manner. Further research is necessary to confirm these associations in larger and more diverse populations and to investigate the potential for chrononutrition strategies to support metabolic health.

**Figure:**
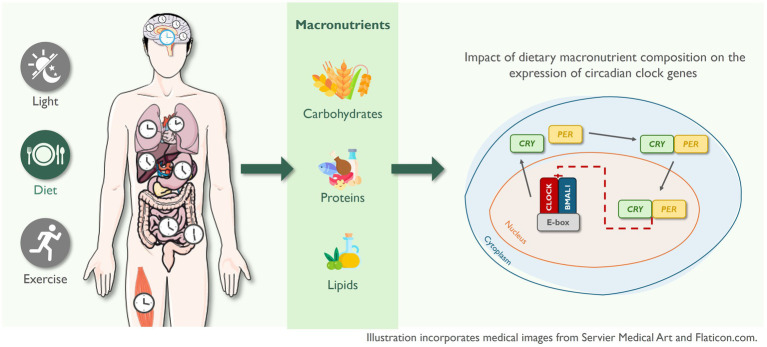

## Introduction

The circadian system is a hierarchical network that is regulated at both the central and peripheral levels by autonomous circadian clocks. The suprachiasmatic nucleus (SCN), located in the anterior part of the hypothalamus (1), constitutes the central clock and functions as the master circadian regulator, synchronizing the peripheral clocks located in nearly all tissues and organs through neurohormonal and metabolic signals, thereby orchestrating systemic circadian rhythms (2–4). The central clock is primarily entrained by the light–dark cycle, while peripheral clocks are more responsive to other external cues such as eating patterns and physical activity (5). At the molecular level, circadian rhythms are governed by a set of core clock genes, including *PER1*, *PER2*, *PER3*, *CRY1*, *REV-ERBα (NR1D1)*, *BMAL1,* and *CLOCK*, which interact through positive and negative transcriptional and translational feedback loops to generate self-sustained oscillations. *CLOCK* and *BMAL1* form a heterodimer that promotes the transcription of negative regulators, including *PER1*, *PER2*, *CRY1,* and *CRY2*. As PER and CRY proteins accumulate, they inhibit their own transcription by suppressing *CLOCK*/*BMAL*1 activity, thereby completing the feedback loop that governs the rhythm of approximately 24 h. Throughout the day, *PER* and *CRY* proteins are degraded within the proteosome, allowing the transcription of *CLOCK*/*BMAL*1 and resetting a new 24-h cycle (6).

These core clock genes are involved in numerous physiological processes, including sleep–wake cycles, glucose and lipid metabolism, hormone secretion, and overall energy homeostasis, and are active in both central and peripheral tissues (6–9). For instance, *BMAL1* regulates lipid metabolism (10), while *PER2* has been associated with glucose metabolism (11). Disruptions in the rhythmic oscillatory expression of these clock genes have been linked to the onset and progression of metabolic disorders, including obesity, highlighting the circadian clock as both a target and mediator of metabolic dysfunction (12–15).

In human peripheral tissues, the expression patterns of circadian genes vary over the course of the day. *CLOCK* tends to show relatively stable expression across the day (16, 17), while *BMAL1* displays robust diurnal oscillations, with peak expression typically occurring in the late night to early morning (16–18). *PER2* usually peaks in the early morning and begins to decline in the afternoon (18, 19), whereas *CRY* genes tend to show peak or sustained elevated expression later in the morning to early afternoon (16, 17). However, these patterns can shift under metabolic or environmental challenges, including altered sleep–wake cycles, changes in metabolic status, or changes in dietary intake, which contribute to some inconsistencies between studies (20, 21).

Peripheral clocks are highly responsive to external cues, with food intake acting as one of the most potent zeitgebers (time-givers) (2). In this context, chrononutrition has emerged as a field dedicated to exploring the interactions between biological rhythms and nutritional behaviors and how their interplay influences health outcomes. Chrononutrition includes key aspects such as the timing and distribution of energy intake, meal frequency (how often meals are consumed throughout the day), regularity (the consistency of meal times from day to day), and the duration of the daily eating and fasting windows (22). Under physiological conditions, food intake is both regulated by and a regulator of circadian rhythm, highlighting a bidirectional relationship that helps maintain endogenous circadian rhythms (23).

While much attention has been given to the timing of food intake, evidence regarding the impact of diet composition on circadian clock gene expression remains limited. However, findings from animal studies suggest that specific macronutrient profiles can alter the expression and rhythmicity of clock genes in a tissue-specific manner, highlighting the need for further investigation in human models. For example, Hatori et al. (24) showed that mice fed a high-fat diet exhibited a decreased amplitude of expression of most clock genes in the liver. Similarly, Goede et al. (25) showed that a high-fat and high-sugar intake induced tissue-specific alterations in clock gene rhythmicity. In their study, mice given *ad libitum* access to both diets (referred to as a free-choice high-fat/high-sugar diet) during the light phase exhibited attenuated or phase-shifted clock gene expression in skeletal muscle, while rhythmicity in brown adipose tissue was amplified.

Given that the impact of dietary macronutrient composition on the expression of circadian clock genes in humans remains largely unexplored, this observational study aimed to explore the association between dietary intake and clock gene expression in the whole blood of individuals with different metabolic profiles.

## Methods

### Ethical statement

This study is part of the NutriClock Project, which has been submitted to the Ethics Commission of the Portuguese Regional Health Administration of the Centre and has received approval on 29 January 2021 (Proc. No.67–2020). The study complies with the ethical principles of the Declaration of Helsinki, adhering to international, European, and national legislation, including the General Data Protection Regulation (Regulation (EU) 2016/679). This study was conducted with adults aged 18 years and older. Only individuals capable of providing informed consent were included. Participants did not receive any financial or other compensation for their participation. The informed consent document included detailed information on the study’s objectives, procedures, potential risks, and benefits, ensuring participants made an informed decision about their involvement in the study.

### Study population

The guidelines of the study protocol have been published (26). Eligibility was assessed during the initial visit, where inclusion and exclusion criteria were evaluated prior to obtaining informed consent.

Participants included adults aged between 18 and 75 years, with a body mass index (BMI) over 18.5 kg/m^2^. Individuals were excluded if they worked nights or rotating shifts, crossed more than two time zones within the 2 weeks before the study, were diagnosed with severe sleep disorders, were pregnant, had electronic medical devices or implants, had an infection within 4 weeks before the first visit, and had regularly used (equal to or greater than weekly) medications, such as wakefulness-promoting agents, sedatives, melatonin or melatonin analogues, probiotics, glucocorticoids, and anti-obesity drugs.

### Anthropometric and body composition assessments

Body weight was determined using a calibrated digital scale (SECA 813), height was recorded with a stadiometer (SECA 213), and waist circumference was measured with a measuring tape (SECA 201). These parameters were measured according to the Portuguese Directorate General of Health guidelines for anthropometric measurements in adults (27). Body mass index (kg/m^2^) was calculated and categorized according to the WHO’s age- and sex-specific BMI cutoffs for adults (28). Body composition was measured using the bioelectrical impedance analysis equipment SECA medical Body Composition Analyser 525.

### Dietary intake using a three-day food diary

Participants were instructed to complete a 3-day food diary to assess short-term dietary intake and were provided with detailed instructions on how to record all foods and beverages consumed, including portion sizes, preparation methods, and time of consumption. The 3-day food diary was used to capture detailed information on macronutrient composition during a period closely aligned with blood sample collection. Additionally, the 3-day duration was selected to minimize the demands on participants while still providing sufficient dietary detail for analysis, as compared to longer protocols such as 7-day records (29–31).

The completed food diaries were reviewed and analyzed by a trained nutritionist to ensure accuracy and consistency in the data entry. When necessary, participants were contacted to clarify unclear or missing information.

The nutrient composition of food items was determined using the Portuguese Food Nutritional Composition Table published by the National Institute of Health Dr. Ricardo Jorge (32). For food items not listed in the national database, nutrient values were obtained from the United States Department of Agriculture (USDA) food composition tables (33).

### Biochemical evaluation

Participants were asked to arrive at 08:00 a.m., after an overnight fast, for blood collection and subsequent analysis. Briefly, blood was drawn into serum tubes for biochemical analyses, the tubes were centrifuged for 10 min at 3,000 rotations per minute (RPM) and the resulting serum was processed in the automatic analyzer Prestige 24i equipment (Tokyo Boeki, Tokyo, Japan) to evaluate the following parameters: glucose, total cholesterol, low-density lipoprotein cholesterol (LDL-C) direct, high-density lipoprotein cholesterol (HDL-C) direct, and triglycerides.

### Gene expression analysis in blood

On the day of enrollment, whole blood samples were collected at 08:00 a.m. and 04:00 p.m. These timepoints were selected to capture variations in peripheral clock gene expression based on a prior human circadian study that showed more pronounced variations in clock genes during the early morning and late afternoon hours (17). Limiting blood collection to two timepoints also considered the logistical feasibility of repeated sampling in free-living participants. This approach aimed to reduce inconvenience and potential discomfort for the participants, while still enabling the study of potential diurnal variation.

At each timepoint, 3 mL of blood was drawn into Tempus™ Blood RNA Tubes (Thermo Fisher Scientific, MA, USA), containing RNA stabilizing reagent to lyse cells and preserve gene expression profiles. The tubes were inverted and stored at −80 °C until extraction. RNA was isolated using the Tempus™ Spin RNA Isolation Kit (Thermo Fisher Scientific) following the manufacturer’s protocol. RNA concentration and purity were assessed with an ND-1000 Nanodrop spectrophotometer (Thermo Fisher Scientific). RNA was reverse transcribed using the iScript Select cDNA Synthesis Kit (Bio-Rad, CA, USA) in accordance with the manufacturer’s instructions. The resulting cDNA was stored at −20 °C until use.

Quantitative real-time PCR (qRT-PCR) was conducted on the Bio-Rad CFX Connect system (Bio-Rad) using SsoAdvanced™ SYBR® Green Supermix (Bio-Rad). Primers were designed with PrimerBlast, synthesized by Invitrogen, and optimized for concentration and annealing temperature. Reactions (10 μL) contained 5 μL of Supermix, 0.5 μL of each primer, and 4 μL of cDNA. No-Template Controls (NTC) and No-Reverse Transcriptase (NoRT) controls were included. Cycling conditions were 95 °C for 30 s, then 45 cycles of 95 °C for 5 s and 56–60 °C for 30 s, followed by a melting curve (65–95 °C). Relative mRNA levels were quantified using the ΔΔCt method, normalized to GAPDH, the most stable housekeeping gene identified via Genorm and NormFinder.

### Statistical analysis

Descriptive statistics were used to summarize participants’ characteristics. Where applicable, data are presented as mean ± standard deviation (SD) or median and interquartile range (IQR), and categorical variables as frequencies and percentages. Continuous variables were assessed for normality using the Shapiro–Wilk test. Depending on the distribution, differences between groups were analyzed using either the independent samples t-tests (for normally distributed data) or the Mann–Whitney U-test (for non-normally distributed data). Comparisons between categorical variables were conducted using crosstabulation analyses, with either Fisher’s exact test or the Fisher–Freeman–Halton exact test, depending on the contingency table dimensions. Spearman’s and Pearson’s correlation coefficients were calculated to explore the associations between dietary intake, clock gene expression, and metabolic parameters.

Statistical analyses were performed using GraphPad Prism (version 10) and IBM SPSS Statistics software (version 29.0). Statistical significance was set at a *p*-value of <0.05.

## Results

This observational study included 19 participants (94.7% female) with a mean age of 43.4 ± 16.05 years (min: 20 years; max: 69 years). Participants were clustered according to their BMI into the healthy group and the overweight/obesity group. The overweight/obesity group showed significantly more comorbidities (*p* < 0.001) and higher BMI, fat mass, waist circumference and visceral adipose tissue (*p* < 0.01) (Table 1). Lipid profiles also differed significantly, with the overweight/obesity group showing higher total cholesterol, LDL-C, and triglycerides (*p* < 0.05), but no difference in HDL-C (*p* = 0.912). No significant differences were found in skeletal muscle mass and glucose levels.

**Table 1:** Sociodemographic and clinical characteristics of the population.

Participant characteristics	Total (*n* = 19)	Overweight group (*n* = 9)	Healthy group (*n* = 10)	*p*-value
Age (years)	43.4 ± 16.05	49.6 ± 16.15	37.9 ± 14.55	0.116^a^
Sex [*n* (%)]				
Male	1 (5.3%)	1 (11.1%)	0 (0.0%)	0.474^b^
Female	18 (94.7%)	8 (88.9%)	10 (100%)	
Education level [*n* (%)]				
Elementary school	4 (21.1%)	3 (33.3%)	1 (10.0%)	
High school	6 (31.6%)	3 (33.3%)	3 (30.0%)	0.359^c^
Higher education	9 (47.4%)	3 (33.3%)	6 (60.0%)	
Occupation [*n* (%)]				
Paid employment	14 (73.7%)	7 (77.8%)	7 (70.0%)	
Student	3 (15.8%)	1 (11.1%)	2 (20.0%)	1.000^c^
Retired	2 (10.5%)	1 (11.1%)	1 (10.0%)	
Comorbidities [*n* (%)]				
0 to 1	12 (63.2%)	2 (22.2%)	10 (100%)	**<0.001**^**b**^
2 or more	7 (36.8%)	7 (77.8%)	0 (0.0%)	
BMI [kg/m^2^]	23.08 [21.30–31.34]	31.34 [27.83–34.88]	21.67 [20.24–22.79]	**<0.001**^**d**^
Fat mass (%)	34.90 [25.90–47.40]	47.40 [38.00–52.20]	27.70 [24.90–30.40]	**<0.001**^**d**^
SMM (kg)	18.35 [17.05–22.63]	18.85 [17.22–24.11]	17.83 [16.20–19.78]	0.191^d^
WC (cm)	73.40 [71.00–100.00]	100.00 [88.00–109.00]	72.50 [65.00–73.00]	**0.001**^d^
VAT (L)	0.70 [0.50–2.50]	2.50 [1.50–3.10]	0.55 [0.40–0.70]	**0.011**^**d**^
Glucose (mg/dL)	86.70 [75.70–91.10]	88.50 [85.50–91.10]	81.95 [74.40–90.90]	0.243^d^
Total cholesterol (mg/dL)	187.4 ± 44.63	215.4 ± 37.88	162.2 ± 34.93	**0.005**^**a**^
HDL cholesterol (mg/dL)	51.3 ± 11.81	51.0 ± 12.82	51.6 ± 11.51	0.912^a^
LDL cholesterol (mg/dL)	119.6 ± 41.53	145.2 ± 39.63	96.6 ± 28.46	**0.007**^**a**^

HDL, high-density lipoprotein; LDL, low-density lipoprotein. Data are presented as frequencies and valid percentages, mean ± SD, or as median [IQR].

a Independent sample *t*-test.

b Fisher’s exact test.

c Fisher–Freeman–Halton exact test.

d Mann–Whitney U-test.

Values shown in bold are statistically significant.

Mean energy intake was assessed using the 3-day food diaries and did not significantly vary between the two BMI groups (Table 2). In terms of macronutrient distribution, only the percentage of energy derived from protein significantly differed between groups, being higher in the overweight/obesity group compared to the healthy-weight group.

**Table 2:** Daily energy intake and percentages of macronutrient distribution.

Dietary components	Total (*n* = 19)	Overweight group (*n* = 8)	Healthy group (*n* = 10)	*p*-value
Energy (kcal)	1662.1 ± 396.45	1640.4 ± 343.13	1679.4 ± 452.28	0.843^a^
Proteins (%)	19.0 ± 3.20	21.2 ± 2.10	17.3 ± 2.88	**0.005**^**a**^
Carbohydrates (%)	45.8 ± 5.47	44.1 ± 5.37	47.1 ± 5.42	0.252^a^
Lipids (%)	32.0 ± 5.65	31.4 ± 5.17	32.4 ± 6.24	0.723^a^
SFA (%)	10.1 [9.25–12.60]	10.1 [7.88–13.30]	10.4 [9.37–11.40]	0.965^b^
MUFA (%)	11.0 ± 2.83	10.1 ± 3.27	11.6 ± 2.39	0.279
PUFA (%)	5.6 [4.22–6.32]	6.0 [5.40–6.63]	5.0 [3.88–5.99]	0.237^b^

Data are presented as mean ± SD or as median [IQR].

a Independent sample *t*-test.

b Mann–Whitney U-test.

Values shown in bold are statistically significant. SFAs, saturated fatty acids; MUFAs, monounsaturated fatty acids; PUFAs, polyunsaturated fatty acids.

Clock gene expression was assessed in whole blood at two distinct time points, 08:00 a.m. and 04:00 p.m. No statistically significant differences in the expression of *CLOCK*, *BMAL1*, *PER2,* and *CRY* were observed between the overweight/obesity and healthy BMI groups (Table 3). Although the expression of *PER2* and *CRY* at 08:00 a.m. was numerically higher in the overweight/obesity group, these differences did not reach statistical significance.

**Table 3:** Circadian clock genes mRNA expression in whole blood.

Timepoints	Total (*n* = 19)	Overweight group (*n* = 8)	Healthy group (*n* = 10)	*p*-value
*CLOCK*				
08:00 a.m.	1.176 ± 1.1368	1.397 ± 1.6595	0.978 ± 0.2096	0.438^a^
04:00 p.m.	0.816 ± 1.1383	0.603 ± 1.6595	1.009 ± 0.2384	0.454^a^
*BMAL1*				
08:00 a.m.	1.038 ± 0.2270	1.055 ± 0.1496	1.028 ± 0.2688	0.839^a^
04:00 p.m.	0.909 [0.7162–1.0946]	0.834 [0.6612–0.9705]	1.045 [0.7966–1.1800]	0.146^b^
*PER2*				
08:00 a.m.	1.479 ± 1.7856	2.144 ± 2.7107	0.980 ± 0.0939	0.341^a^
04:00 p.m.	0.533 ± 1.7856	−0.053 ± 2.4815	0.989 ± 0.1292	0.309^a^
*CRY*				
08:00 a.m.	1.114 ± 0.4730	1.509 ± 0.8459	0.965 ± 0.1516	0.381^a^
04:00 p.m.	1.102 ± 0.6524	1.256 ± 0.9179	0.964 ± 0.2395	0.378^a^

Data are presented as mean ± SD or as median [IQR].

a Independent sample *t*-test.

b Mann–Whitney U-test.

Correlation analyses revealed multiple statistically significant associations between core clock gene expression and macronutrient intake, with differences observed between healthy-weight and overweight groups (Table 4). In the healthy BMI group, *BMAL1* expression at 08:00 a.m. showed a strong positive correlation with total lipid intake and saturated fatty acid (SFA) intake, whereas at 04:00 p.m., it was inversely correlated with SFA intake. *CLOCK* expression at 04:00 p.m. correlated negatively with lipid intake. *CRY* expression at 08:00 a.m. was positively correlated with monounsaturated fatty acid (MUFA) intake, while the expression at 04:00 p.m. was negatively correlated with lipid intake and positively correlated with protein intake. In the overweight/obesity group, *CLOCK* expression at 08:00 a.m. was inversely correlated with both lipid and carbohydrate intake, whereas the expression at 04:00 p.m. was positively correlated with both lipid and carbohydrate intake.

**Table 4:** Associations between macronutrient intake and clock gene expression in whole blood.

Dietary components	*BMAL1*		*CLOCK*		*PER2*		*CRY*	
	8 a.m.	4 p.m.	8 a.m.	4 p.m.	8 a.m.	4 p.m.	8 a.m.	4 p.m.
Lipids (%)								
Overweight	*r* = 0.358	*ρ* = −0.036	*r* = −0.754*	*r* = 0.754*	*r* = −0.737	*r* = 0.761	*r* = −0.826	*r* = 0.514
Healthy	*r* = 0.678*	*ρ* = −0.467	*r* = 0.560	*r* = −0.670*	*r* = 0.037	*r* = 0.053	*r* = 0.316	*r* = −0.793*
SFA (%)								
Overweight	*ρ* = −0.100	*ρ* = 0.464	*ρ* = −0.429	*ρ* = 0.429	*ρ* = −0.086	*ρ* = 0.086	*ρ* = −0.500	*ρ* = 0.429
Healthy	*ρ* = 0.733*	*ρ* = −0.709*	*ρ* = 0.139	*ρ* = −0.176	*ρ* = −0.143	*ρ* = 0.150	*ρ* = −0.286	*ρ* = −0.176
MUFA (%)								
Overweight	*r* = 0.087	*ρ* = 0.107	*r* = −0.705	*r* = 0.705	*r* = −0.636	*r* = 0.652	*r* = −0.628	*r* = 0.554
Healthy	*r* = −0.516	*ρ* = 0.127	*r* = 0.543	*r* = −0.442	*r* = 0.366	*r* = −0.087	*r* = 0.760*	*r* = −0.205
PUFA (%)								
Overweight	*ρ* = 0.200	*ρ* = −0.214	*ρ* = −0.571	*ρ* = 0.571	*ρ* = −0.086	*ρ* = 0.371	*ρ* = −0.143	*ρ* = 0.214
Healthy	*ρ* = −0.250	*ρ* = 0.418	*ρ* = 0.139	*ρ* = −0.055	*ρ* = 0.286	*ρ* = −0.400	*ρ* = −0.143	*ρ* = 0.067
Proteins (%)								
Overweight	*r* = −0.384	*ρ* = −0.179	*r* = −0.252	*r* = 0.252	*r* = −0.202	*r* = 0.196	*r* = −0.240	*r* = −0.075
Healthy	*r* = −0.540	*ρ* = 0.139	*r* = −0.091	*r* = 0.225	*r* = −0.161	*r* = 0.235	*r* = −0.062	*r* = 0.663*
CH (%)								
Overweight	*r* = −0.163	*ρ* = 0.143	*r* = −0.794*	*r* = −0.794*	*r* = 0.748	*r* = −0.711	*r* = 0.741	*r* = −0.511
Healthy	*r* = −0.428	*ρ* = 0.479	*r* = −0.564	*r* = 0.603	*r* = 0.109	*r* = −0.190	*r* = −0.145	*r* = 0.466

SFAs, saturated fatty acids; MUFAs, monounsaturated fatty acids; PUFAs, polyunsaturated fatty acids; CH, carbohydrates. *p < 0.05. Values shown in bold are statistically significant.

Additionally, correlation analyses were conducted to explore associations between clock gene expression, dietary intake, and metabolic parameters. A significant negative correlation was found between *BMAL1* expression at 04:00 p.m. and LDL-C levels (*ρ* = −0.486, *p* = 0.041). Moreover, a positive correlation was observed between the percentage of dietary protein intake and triglyceride levels (*r* = 0.655, *p* = 0.003). No other significant correlations were found between clock gene expression, dietary intake, and metabolic parameters.

## Discussion

This study underscores the intricate interactions between dietary intake and the regulation of circadian genes. Macronutrient distribution, particularly lipid intake, was associated with time-dependent variations in clock gene expression in whole blood, suggesting that both nutrient composition and timing may influence peripheral clock regulation.

Circadian disruption has been increasingly associated with metabolic disturbances in humans, including obesity (34–36). Irregular sleep and eating patterns, commonly observed in shift workers, can desynchronize endogenous circadian rhythms and metabolic processes, impairing the temporal regulation of metabolic pathways and promoting weight gain and metabolic dysregulation (35, 37, 38). These associations have driven interest in circadian-based interventions, including chrononutrition, an approach that aligns meal timing and composition with biological rhythms to optimize metabolic outcomes.

Evidence suggests that the molecular components of the circadian clock are responsive to food intake (39, 40). However, the specific influence of macronutrient composition on clock gene expression in humans remains poorly understood. In this study, significant associations were observed between the expression of clock genes and the relative intake of lipids, including SFA and MUFA, proteins, and carbohydrates. These associations varied between the morning and afternoon points, suggesting that both the timing and macronutrient profile of the diet may modulate the expression of circadian genes.

Our findings align with evidence from animal studies, which have shown that feeding can reset peripheral clocks, particularly in the liver (41, 42). For example, Hirao et al. (43) reported that only a combination of carbohydrates and proteins, and not individual macronutrients alone, could effectively reset hepatic circadian rhythms in mice. Oike et al. (44) similarly showed that the intraperitoneal administration of glucose with amino acids, but not each nutrient alone, resets hepatic clocks. Furthermore, insulin has been shown to acutely induce *PER1*, *PER2*, and *DEC1* expression in hepatic cells, implicating nutrient-sensitive hormonal signaling in peripheral clock entrainment (45). While our study assessed gene expression in whole blood rather than the liver, the observed associations suggest that similar mechanisms may influence human peripheral tissues.

Beyond protein and carbohydrate intake, dietary lipids also appear to play a significant role in circadian regulation. Lipid metabolism and circadian rhythms are closely linked, with growing evidence underscoring their bidirectional influence. Core clock genes, such as *CLOCK* and *BMAL1*, regulate lipid synthesis, degradation, and transporter expression (46–48). In our study, the healthy BMI group showed significant associations between *BMAL1* expression and the percentage of total lipid intake at 08:00 a.m., as well as with the percentage of SFA intake at both 08:00 a.m. and 04:00 p.m. In contrast, no such associations were detected in the overweight/obesity group. This finding may indicate a preserved responsiveness of peripheral clocks to dietary lipid cues in metabolically healthy individuals, potentially supporting circadian alignment between nutrient availability and metabolic gene expression. In contrast, the absence of association in the overweight/obesity group may reflect impaired nutrient sensing or circadian desynchronization, phenomena often linked to metabolic dysfunction.

The significant negative correlation found between *BMAL1* expression in the afternoon and LDL-C levels reinforces the potential link between disrupted circadian gene expression and adverse lipid profiles, as shown before in preclinical studies. Pan and Hussain (46) showed that *BMAL1* influences lipoprotein synthesis and maturation in Western diet-fed mice, while Gu et al. (49) found hepatocyte-specific *BMAL1* knockout disrupted hepatic circadian rhythms, resulting in enhanced lipid catabolism alongside reduced lipid synthesis and fatty acid oxidation. Moreover, high-fat diets have been found to dampen clock gene expression rhythms in liver and adipose tissue (50) and induce phase advances in liver circadian clocks (51, 52). Mice fed a ketogenic diet displayed similar advances in the phase of *PER2* and other clock-controlled genes expression in peripheral tissues (53), reinforcing the idea that dietary lipids can serve as both substrates and circadian modulators. However, the extent to which these findings apply to humans remains unclear, and the effects of specific dietary patterns, such as high-protein, high-fat, or ketogenic diets, on circadian gene expression in human tissues have not been conclusively established.

While lipids appear to influence clock gene regulation, particularly *BMAL1*, and may be linked to lipid-related biomarkers such as LDL-C, other macronutrients may impact distinct metabolic pathways. For instance, protein intake was found to positively correlate with triglyceride levels. Although high-protein diets are frequently associated with metabolic benefits, excessive protein intake, particularly from red meat or processed sources, may lead to elevated triglyceride concentration (54). This result also aligns with mechanistic evidence from a controlled trial that showed that, in healthy individuals, a high-protein diet increased *de novo* lipogenesis-associated triglyceride production, particularly when the protein is glutamate-rich (55). However, in our sample, individuals with higher protein intake were predominantly from the overweight/obesity group, who typically present altered metabolic profiles (56). This group distribution may have contributed to the observed positive correlation between protein intake and triglyceride levels.

Clock gene expression in human adipose tissue has also been linked to metabolic status. Vieira et al. (57) found increased *CRY2* and *REV-ERBα* expression in the visceral adipose tissue of women with obesity, while Straczkowski et al. (58) reported reduced *REV-ERBα*, *REV-ERBβ*, *PER1*, and *PER2* expression in individuals with overweight/obesity compared to healthy-weight individuals. In our study, although no statistically significant differences were observed in clock gene expression between BMI groups, group-level variations suggest differential circadian regulation. For example, *CLOCK* expression showed greater amplitude between time points in the overweight/obesity group, whereas more stable expression was observed in the healthy BMI group. This greater variability may indicate circadian misalignment or weakened peripheral rhythmicity in individuals with higher adiposity, consistent with reports that *CLOCK* expression tends to be more stable in healthy individuals (16). Such instability could be driven by obesity-associated factors such as insulin resistance, low-grade inflammation, or disrupted hormonal signaling, which are known to interfere with circadian gene feedback loops (59).

Furthermore, the overweight/obesity group exhibited numerically higher morning *PER2* levels and lower afternoon levels compared to the healthy BMI group, though these differences did not reach statistical significance. This pattern may reflect altered circadian regulation, consistent with findings from rodent models where post-fasting refeeding, particularly meals rich in protein and carbohydrates, induced rapid shifts in liver clock gene expression, including *PER2* (60). The higher protein intake in the overweight/obesity group may contribute to this trend, but metabolic impairments common in obesity, such as insulin resistance, may blunt or dysregulate nutrient-induced clock resetting. This finding suggests that the same dietary input might elicit distinct circadian responses depending on metabolic health: in healthy individuals, nutrients may reinforce rhythmicity, while in metabolically compromised individuals, responses may be blunted or misaligned.

However, despite these apparent trends, the lack of significant differences in clock gene expression between BMI groups might not only be limited to the small sample size but also to other biological and methodological factors. Gene expression was measured in whole blood, not capturing the activity of circadian clocks in metabolically active tissues such as liver, adipose tissue, or skeletal muscle, where obesity-related alterations may be more pronounced (61). Next, our sampling strategy involved only two time points for blood collection, which may not sufficiently depict circadian oscillations or detect potential phase shifts in gene expression. Circadian misalignment may manifest through altered timing or amplitude, which requires higher temporal resolution to observe. Inter-individual variability related to chronotype, dietary habits, sleep–wake behaviors, or hormonal status could also hamper group-level differences. Finally, BMI alone does not necessarily reflect metabolic health status. Participants within the same BMI group may exhibit distinct phenotypes despite similar body weight classifications (62).

Skeletal muscle mass is a key component of body composition and may also contribute to variability in responses between individuals. However, no significant differences were observed between BMI groups in this variable. This may result from the relatively young to middle-aged, apparently healthy status of the participants, in whom pronounced muscle loss is unlikely. Research suggests that skeletal muscle is not only a key metabolic tissue but also presents its own circadian clock, which may influence muscle function and metabolism (63). While our study did not examine muscle-specific clock gene expression, the role of circadian rhythms in regulating muscle physiology may warrant further research, particularly in relation to metabolic health and body composition profiles.

While hormonal status was not assessed in this pilot study, it is important to note that hormonal fluctuations related to the perimenopausal transition may also have influenced the observed patterns of clock gene expression. The majority of participants in this study were women with a mean age of 43.4 years, an age range that commonly overlaps with perimenopause. Among women in this age range, fluctuations in hormones can affect circadian regulation, as receptors for these hormones are expressed in the SCN and in various peripheral tissues (64, 65). Sex steroids have a direct impact on circadian rhythms, while estradiol advances and progesterone delays circadian rhythms (66), contributing to multiple metabolic and sleep-related alterations observed in perimenopause. The hormonal cycles are also under circadian regulation; therefore, their direct association is unquestionable (67). Moreover, estradiol has been shown to modulate the expression of *PER2* in rats (68).

The findings of this pilot study support the idea that, together, nutrient composition, timing of food intake, and metabolic phenotype shape the circadian clock, with implications for personalized chrononutrition. Increasing evidence suggests that both diet composition and time play crucial roles in modulating metabolic rhythms and influencing health outcomes. For instance, Lundell et al. (69) demonstrated that time-restricted eating (TRE) enhanced the rhythmicity, particularly the amplitude and phase, of serum lipids and skeletal muscle metabolites, without altering the expression of core clock genes in skeletal muscle. Lipids represented the largest class of oscillating serum metabolites, underscoring the powerful role of meal timing in regulating lipid metabolism independently of molecular clock gene expression.

Some limitations should be considered when interpreting the results of this study. First, the sample exhibited limited sex diversity, with only one male participant. This imbalance resulted from recruitment challenges during the early stages of the ongoing study. Women were generally more receptive to participating, which contributed to the observed sex imbalance. Given that this study was included within the NutriClock research project, and considering evidence from recent research indicating that women are more available and willing to participate in studies within the chrononutrition field (70), it is likely that our expanded sample will continue to show a predominance of female participants. The small sample size may also limit the statistical power and the generalizability of the findings. As described in the study protocol (22), the sample size was calculated to include a minimum of 23 participants in each group in order to detect significant differences in clock gene expression, with a significance level of 5% and statistical power of 80%. This brief report presents preliminary data from a database still under development, as the study is in its initial phase. Consequently, the analysis was based on a smaller subset of participants, which may limit the robustness and external validity of the findings. Additionally, due to the small sample size, it was not feasible to perform multivariate regression models adjusting for potential confounding factors such as age, BMI, or metabolic markers. As these variables influence both dietary intake and circadian gene expression, future studies with larger and more diverse samples should incorporate these statistical adjustments.

Furthermore, although food diaries are a widely used method for assessing short-term dietary intake, several limitations need to be acknowledged in their application. Incomplete or missing entries potentially lead to under- or overestimation of the actual intake. In some cases, participants failed to report precise quantities consumed, requiring the use of standard portion sizes, which may not accurately reflect individual eating behaviors and portion preferences. This might introduce measurement errors and affect the accuracy of nutrient estimates. Moreover, social desirability bias may influence participants to alter their reported intake, either consciously or unconsciously, to conform to perceived dietary norms, and the act of recording food intake can itself lead to changes in eating behavior (71, 72).

To address current limitations and advance the understanding of the relationship between circadian biology, diet, and metabolism, future studies should prioritize larger and more diverse populations to ensure broader representation and generalizability. Comprehensive sampling protocols are also needed to more accurately capture circadian fluctuations in gene expression and associated biomarkers. In addition, the use of objective dietary assessment methods, such as weighed food records or digital dietary tracking, would improve the accuracy and reliability of nutritional data. Importantly, longitudinal studies are necessary to determine how dietary intake, particularly diet composition, influences the regulation of the circadian system. Such insights are essential for informing the design of future nutritional interventions that incorporate circadian parameters to enhance their efficacy and metabolic outcomes.

Despite these limitations, this study shows relevant strengths, including the inclusion of participants spanning a broad age range and exhibiting diverse metabolic phenotypes. Moreover, the integrative assessment of circadian clock gene expression combined with detailed dietary intake provided a comprehensive understanding of the interaction between molecular circadian regulation and chronobiological preferences, which might be a determinant for future personalized strategies using chrononutrition.

The present preliminary findings reinforce the growing body of evidence indicating that diet composition interacts with circadian gene expression. Our results suggest that specific macronutrients may modulate peripheral clock gene expression in a time-dependent manner, highlighting molecular-level implications of dietary patterns and supporting the concept that chrononutrition strategies should integrate both nutrient composition and meal timing. However, the broader dietary implications of these associations have yet to be fully elucidated. This underscores the need for further research to address current limitations and to explore how daily dietary intake, including meal timing (e.g., breakfast vs. dinner), macronutrient composition, and nutrient quality, can be optimized to ensure circadian alignment and metabolic health. Well-designed, controlled longitudinal studies will be essential to determine the causal nature of these relationships and to inform the development of evidence-based chrononutrition strategies applicable to diverse populations.

## Conclusion

Circadian clocks play a central role in regulating energy homeostasis and are influenced by both the timing and composition of dietary intake. Building on this connection, this study provides novel insights into the intricate interplay between the molecular circadian clock, dietary intake, and metabolic health. The observed time-dependent associations between macronutrient composition and clock gene expression suggest that both the quality and timing of dietary intake may play a role in modulating the circadian clock. These findings highlight the growing relevance of chrononutrition as a strategy to optimize metabolic health by synchronizing dietary habits with the body’s internal clock. To further clarify these relationships and translate them into practical guidance, future research, particularly longitudinal and well-controlled interventional studies, is needed to investigate the causal effects of dietary patterns on circadian gene expression and associated health outcomes.

## Data Availability

The datasets presented in this article are not readily available because the study is ongoing and the qPCR dataset is preliminary and incomplete. Requests to access the datasets should be directed to sara.silva@estescoimbra.pt.
