# Correction: Association between dietary intake and the expression of clock genes in adults: a brief report

**DOI:** 10.3389/fnut.2026.1747601

**Published:** 2026-01-21

**Authors:** Marlene Lages, Renata Barros, Marisa Ferreira-Marques, Joana Correia, Armando Caseiro, Maria P. Guarino, Sara Carmo-Silva

**Affiliations:** 1 ciTechCare – Center for Innovative Care and Health Technology, Polytechnic University of Leiria, Leiria, Portugal; 2 Faculty of Nutrition and Food Science, University of Porto, Porto, Portugal; 3 Laboratory for Integrative and Translational Research in Population Health (ITR), University of Porto, Porto, Portugal; 4 EPIUnit – Institute of Public Health, University of Porto, Porto, Portugal; 5 Coimbra Health School, Polytechnic University of Coimbra, Rua da Misericórdia, Lagar dos Cortiços, S. Martinho do Bispo, Coimbra, Portugal; 6 CNC-UC – Center for Neuroscience and Cell Biology, University of Coimbra, Coimbra, Portugal; 7 CIBB – Center for Innovation in Biomedicine and Biotechnology, University of Coimbra, Coimbra, Portugal; 8 Faculty of Pharmacy, University of Coimbra, Coimbra, Portugal; 9 H&TRC- Health and Technology Research Center, Coimbra Health School, Polytechnic University of Coimbra, Rua 5 de Outubro, Coimbra, Portugal; 10 University of Coimbra, CIPER, Coimbra, Portugal; 11 ESSLei, School of Health Sciences, Polytechnic University of Leiria, Leiria, Portugal

**Keywords:** chrononutrition, circadian rhythms, diet, obesity, clock genes

There was an error in the last sentence of the section **Results**, Paragraph One:

The sentence read as “ No significant differences were found in skeletal muscle mass or waist circumference.” The corrected paragraph appears below.

“This observational study included 19 participants (94.7% female) with a mean age of 43.4 ± 16.05 years (min: 20 years; max: 69 years). Participants were clustered according to their BMI into the healthy group and the overweight/obesity group. The overweight/obesity group showed significantly more comorbidities (*p* < 0.001) and higher BMI, fat mass, waist circumference and visceral adipose tissue (*p* < 0.01) (Table 1). Lipid profiles also differed significantly, with the overweight/obesity group showing higher total cholesterol, LDL-C, and triglycerides (*p* < 0.05), but no difference in HDL-C (*p* = 0.912). No significant differences were found in skeletal muscle mass and glucose levels.”

The original version of this article has been updated.

